# Development and Evaluation of a Novel Curriculum for Whole Blood Transfusion by Paramedics in the Prehospital Environment

**DOI:** 10.5811/westjem.19438

**Published:** 2025-05-13

**Authors:** Eric Garfinkel, Robby May, Asa Margolis, Eric Cohn, Steven Colburn, Tom Grawey, Matthew Levy

**Affiliations:** * Johns Hopkins University, Department of Emergency Medicine, Baltimore, Maryland; † Howard County, Department of Fire & Rescue Services, Marriottsville, Maryland; ‡ Medical College of Wisconsin, Department of Emergency Medicine, Milwaukee, Wisconsin

## Abstract

**Introduction:**

Resuscitation with low-titer O+ whole blood improves the outcomes of patients with hemorrhagic shock. Recently, some emergency medical services (EMS) agencies have started to carry blood in the field. However, there exists no standardized training program to teach paramedics the fundamentals of blood administration. This study describes one EMS system’s experience with implementing a novel, whole blood educational curriculum.

**Methods:**

We used Kern’s six-step framework to develop a novel curriculum to provide paramedics the requisite knowledge to safely administer blood in the field. The course included an asynchronous component as well as an in-person, skills competency verification. The asynchronous portion was open to any paramedic, but only paramedic supervisors were eligible for the in-person skills check as they are the ones tasked with administering blood in the field. The course was evaluated through survey and performance outcome measurements.

**Results:**

Fifty-three (26.5%) of 200 total paramedics at a combined career and volunteer fire department enrolled in the asynchronous course, and 31 (58.5%) completed the pre- and post-course survey. Of participating paramedic supervisors, 20 of 20 (100%) finished both portions of the course. Survey answers were based on a 5-point Likert scale. We reported results as a mean, with 5 corresponding to “strongly comfortable” or “strongly agree.” There was a statistically significant increase in the number of respondents who felt overall comfortable in administering blood from 3.51 to 4.16 (*P* = 0.003). Additionally, there was an increase in the number of paramedics who reported feeling comfortable performing the procedure of a blood transfusion from 3.11 to 4.13 (*P* = <0.001). Nearly all participants (30/31) would recommend the course to someone else. In the first three months of carrying blood in the field, there were 12 units of blood transfused and no protocol deviations or safety events.

**Conclusion:**

This study provides a model for the successful creation and implementation of a prehospital blood transfusion educational program using Kern’s framework. The curriculum was implemented in a single EMS system with senior paramedics, which may limit generalizability.

## INTRODUCTION

Early resuscitation of hemorrhagic shock with blood products improves patient outcomes.^1^–^6^ In 2017, two Texas emergency medical service (EMS) agencies became the first civilian ground EMS systems in the United States to carry low-titer O+ whole blood (LTO+WB).^7^ The success of these programs and others across the country demonstrated the value of a prehospital whole blood program.^8^ As of August 2023, there are over 112 active ground-based EMS whole blood programs.^9^

Emergency medical services clinicians are trained to recognize hemorrhagic shock and initiate life-saving measures, such as a tourniquet or wound packing.^10^,^11^ Blood administration, however, is not currently a mandatory topic in initial paramedic training.^10^ As paramedics are increasingly tasked with transfusing blood in the field, there is a need for standardized supplementary training to ensure competency in initiating a blood transfusion and managing potential complications.

In September 2023 the Howard County Department of Fire and Rescue Services (HCDFRS) became Maryland’s first EMS ground agency to carry LTO+WB. In this paper, we describe the development of a curriculum to teach paramedics the key components of prehospital whole blood transfusion. Our goal was to provide a framework for other EMS agencies that are creating a whole blood education program.

## METHODS

### Study Design

This is a descriptive study of the curriculum development process and a retrospective review of survey data obtained from course participants as a part of evaluation and feedback. The Johns Hopkins University School of Medicine Institutional Review Board approved this study as exempt research.

### Study Setting and Population

We conducted this study at HCDFRS in Howard County, Maryland. The department responds to over 30,000 calls annually and has approximately 200 paramedics. At any given time, three medical duty officers (MDO) are stationed in the county. The MDO is an experienced paramedic with at least five years of EMS experience, including a minimum of two years as an Advanced Life Support (ALS) responder. An MDO is dispatched to all high-acuity calls and provides advanced capabilities such as rapid sequence intubation and whole blood administration. A minimum of one unit of LTO+WB is carried in each MDO vehicle. There are 20 MDOs in the department, all of whom were required to take the blood administration training course.

### Curriculum development

The HCDFRS LTO+WB curriculum was created in part as a capstone project for the EMS Educator’s Collaborative, sponsored by the National Association of EMS Physicians and the National Association of EMS Educators. We used Kern’s six-step framework for this project.

#### I. Problem identification and general needs assessment

No ground EMS service in the state of Maryland was carrying blood products at the time that the HCDFRS whole blood program was created; therefore, no educational programs existed that incorporated the specifics of the Maryland state protocol and unique challenges of ground EMS. Thus, there was an essential need to develop a novel curriculum to ensure HCDFRS paramedics could transfuse blood safely and appropriately to patients in hemorrhagic shock.

#### II. Targeted needs assessment

Knowledge of blood transfusion complications and maintaining a blood transfusion are expected competencies in the 2021 National EMS Education Standards and the 2019 National EMS Scope of Practice, respectively.^10^,^11^ However, neither document refers to initiating a blood transfusion. Knowledge of the indications and contraindications for LTO+WB is essential and is not routinely covered in the paramedic curriculum. Additionally, the MDOs in our study averaged a decade since initial paramedic schooling and had no bedside experience in blood transfusion. A literature review revealed only one publicly available comprehensive training curriculum whose target audience included paramedics. This program, created by the Trauma Hemostasis and Oxygenation Research Network, provides an excellent overview but does not provide the depth of content necessary to cover all objectives nor does it address the nuances of specific state and departmental protocols.^12^ Thus, the creation of a robust training program, novel due to its focus specifically on prehospital care, was necessary.

#### III. Goals and objectives

The goal of the curriculum was for paramedics to demonstrate the key components of prehospital blood transfusion and possess competency in the requisite knowledge to administer blood to a critically ill patient in the prehospital environment. We created objectives using Bloom’s taxonomy framework (Table 1). Consensus on the goals and objectives was obtained from board-certified EMS physicians at HCDFRS, departmental leadership, professional paramedic educators, and the Maryland State EMS medical director.

#### IV. Educational strategies

To ensure flexibility and maximize efficiency, a portion of the curriculum was delivered asynchronously using the department’s online learning management system. Competency was assessed during an in-person skills check.

#### V. Implementation

Two EMS physicians (EG, ML), a doctorate-prepared EMS educator (RM), and a HCDFRS EMS captain (EC), created nine, narrated PowerPoint modules spanning 134 minutes (Table 1). Each module contained a brief formative assessment to ensure understanding. The in-person component included a review of the asynchronous content followed by a skills check. This was scheduled for three hours with a group of up to 10 learners (Table 1). The EMS physicians and an EC performed the review and skill checks. Completion of the online portion was required before attending the in-person session.

Development of the course, plus implementation of the asynchronous content, required approximately 80 hours of work. Half of this time was from EMS physicians, and the other half was from EMS educators. Four in-person were held, each of which were three hours in length. The in-person classes included a physician and EMS educator. Although exact numbers are difficult to obtain and costs will vary substantially between departments, we estimate the total cost for HCDFRS of designing, implementing, and executing this program was ≈$14,960.

#### V1. Evaluation

Before- and after-surveys were distributed to assess reaction level data. Program effectiveness is measured at the behavior and outcome levels through review of every blood administration in the field. Protocol compliance and patient outcomes are determined for each blood transfusion event through 100% case review by an EMS physician medical director. This review includes a debrief with the treating paramedic within 24 hours of the call, review of the prehospital patient care report, and chart review of the hospital course.

Semi-annually a report is generated to identify any patients who met vital sign criteria for blood. Review of the narratives by the EMS physician medical director is performed to determine whether any patient was a candidate for blood but was not transfused.

All protocol deviations and patient complications are documented and handled per departmental policy. All calls involving the use of blood are reviewed during a monthly MDO meeting. The session is led by an EMS physician medical director with input from the MDO who administered the blood.

## RESULTS

The online course was available to all HCDFRS paramedics and mandatory for MDOs. Fifty-three paramedics enrolled in the online course, including the pre-course survey. Thirty-one completed the post-course survey, which was embedded within the online course. All 20 HCDFRS MDOs completed the asynchronous component and attended the hands-on skills session.

We calculated the mean with 95% confidence intervals. Statistical significance was calculated using a two-sample Wilcoxon rank-sum test. Data is reported on a 1–5 scale, with 1 corresponding to “very uncomfortable” or “strongly disagree” and 5 representing “very” comfortable or “strongly agree.” A mean of three correlates to neither comfortable nor uncomfortable or neither agree nor disagree.

Table 2 details the survey results. Among those who took the pre-course survey, 94.3% (50/53) had never administered blood in the field. Most students entered the course strongly agreeing that carrying blood in the field is important (mean 4.21) and safe (mean 4.08), which increased to a mean of 4.45 (*P* = 0.236) and 4.39 (*P* = 0.064), respectively. The comfort level of administering blood increased from a mean of 3.51 to 4.16 (*P* = 0.003). Comfort with the knowledge of the indications (mean = 3.53) and contraindications (mean = 3.19) increased significantly to 4.19 (*P* = 0.001) and 4.16 (*P* = <0.001). Confidence in performing the procedure was neutral before the course (mean = 3.11) and increased afterward (mean = 4.13, *P* = <0.001). Respondents initially felt neutral with pediatric blood transfusion (mean = 3.04) and increased to 3.97 (*P* = <0.001).

Obtaining verbal consent for blood transfusion was initially a comfort level of 3.77 and increased to 4.19 (*P* = 0.045). Comfort with handling a situation in which the patient refused blood due to religious beliefs was 3.77 to 4.19 (*P* = 0.045). Finally, integrating whole blood into the typical care of a patient with hemorrhagic shock was 3.60 followed by 3.94 (*P* = 0.072). The majority (90%, 28/31) felt the course length was just right, and 97% (30/31) would recommend the course.

Ten patients were transfused a total of 12 units of blood in the first six months of the LTO+WB program, which is approximately two patients per month. No protocol deviations or adverse reactions occurred. All patients eligible for blood during this time were correctly identified and transfused. Among the patients who received blood, 60% (6/10) were alive at emergency department arrival and 50% (5/10) were alive at 24 hours. The majority (8/10) were patients with hemorrhagic shock secondary to trauma.

## DISCUSSION

As out-of-hospital transfusion of blood continues to gain popularity across the United States, there is an emerging need to provide paramedics with the knowledge of when and how to initiate a transfusion. Although there are publications regarding best practices of whole blood transfusion in other clinical environments, none exist that focus on blood transfusion by paramedics.^13^,^14^ We aimed to share our experience creating a curriculum to address the needs of an EMS system, with the understanding that many systems across the country share the same barriers.

Kern’s framework ensured training was created that was targeted toward our system’s needs and appropriately accomplished our learning objectives. The hybrid model of using asynchronous narrated lectures plus an in-person skills check allowed for maximal flexibility and limited the need for additional staffing. Further, it has the advantage of being easily distributed to other EMS clinicians within the department and other organizations.

The survey data results suggest that the course successfully achieved its goals, as the mean response to feeling confident that they could identify the correct patient and perform the procedure was 4.19 and 4.13, which corresponds to “agree” on the Likert scale. Notably, this was a statistically significant increase from the pre-course survey. This confidence aligns with our performance data, which demonstrated 100% protocol compliance over the first three months and no safety events. The survey area with the lowest post-course confidence was pediatric blood transfusions (mean = 3.97), which is an area that may need additional explanation in future iterations.

As educators and EMS leaders consider the future of prehospital care, there must be a continued emphasis on development of educational programs that teach paramedics new skills not covered in initial training programs. More departments will likely expect their paramedics to administer blood in the field. This study creates a blueprint and foundation for future learning objectives and educational strategies in developing new curricula for paramedics using the Kern framework.

## LIMITATIONS

This program fulfilled the needs of a specific EMS agency, which limits generalizability. However, many aspects of this program are universal to all patients in hemorrhagic shock. Additionally, the audience for this program was senior paramedic supervisors. The course may need modification for novice EMS responders. Not all participants finished the final survey, which adds potential bias. No complications or protocol deviations occurred; however, the number of patients treated was small, which limits our ability to make any firm conclusions. Finally, further research is required to determine the degree of knowledge retention using this educational approach.

## CONCLUSION

This study describes the creation of a prehospital blood transfusion educational curriculum targeted at paramedic supervisors. Confidence increased in all categories that were surveyed, and no safety events occurred after blood was deployed in the field, although this result was limited by the small sample size. As more EMS agencies carry blood in the field, there will be an increasing demand for this type of content. This curriculum provides a framework for future educational programs.

## Figures and Tables

**Table 1:** Objectives of course in prehospital whole blood transfusion.

Course Objectives by Module
I. Module 1 *(Introduction and Blood Physiology, 35 minutes)* Review the research on the need for whole blood in the prehospital setting Review data of EMS agencies that are currently using whole blood Explain blood type and Rh factor physiology Explain the components of low-titer O+ (LTO+) whole blood (WB) Explain the importance of prehospital blood transfusion II. Module 2 *(Management of the Severely Injured Trauma Patient, 22 minutes)* Compare how management of the trauma patient has changed from the 1990s to today Explain the lethal diamond and the role that LTO+WB has in this patient Review the Multiple Severe Trauma Protocol Review the use of air transport and when it should be activated III. Module 3 *(Indications and Protocol, 22 minutes)* List the indications for administration of LTO+WB List the contraindications for administration of LTO+WB Explain shock index and demonstrate how to calculate it Explain the management of a patient who is having a transfusion reaction IV. Module 4 *(Blood Transfusion in the Medical Patient, 8 minutes)* Review situation in which medical patients may benefit from blood Discuss risk factors that can increase the likelihood of developing hemorrhagic shock V. Module 5 *(Management of the Patient Receiving Blood, 12 minutes)* Explain the safety profile for the use of whole blood in the field Describe the set-up that is needed for the administration of LTO+WB Understand the importance of administration of tranexamic acid and calcium when their administration is appropriate VI. Module 6 *(Ethical Considerations and Consent, 4 minutes)* Review the consent process, including implied consent Discuss patients who may refuse to receive blood products VII. Module 7 *(Special Populations, 8 minutes)* Discuss blood transfusion in pediatrics and women of childbearing age Review management of postpartum hemorrhage VIII. Module 8 *(Cold Chain and Administration, 15 minutes)* Review the equipment needed for cold-chain storage Review the cold-chain storage workflow IX. Module 9 *(Review, 8 minutes)* Review key components of whole blood administration in the field X. In Person Skills Check *(2 hours)* Demonstrate the process of administering blood Assess situations where blood transfusion is appropriate Manage a patient with a transfusion reaction Obtain consent from a patient

*EMS*, emergency medical services.; *LTO+WB*, low-titer O+ whole blood.

**Table 2:** Survey responses.

Survey Question	Pre-Course [n=53]	Post-Course [n=31]	*P*-Value
Have you ever administered blood in the field? [Yes/No]	Yes 5.6% [3/53] No 94.3% [50/53]	---	---
Do you think that carrying blood in the field is important?	Mean 4.21 CI 3.98–4.44	Mean 4.45 CI 4.21–4.70	0.24
Do you think that prehospital blood administration is safe?	Mean 4.08 CI 3.87–4.28	Mean 4.39 CI 4.13–4.64	0.06
Overall, how comfortable are you with whole blood administration in the field?	Mean 3.51 CI 3.22–3.79	Mean 4.16 CI 3.80–4.52	0.003
How comfortable are you knowing the indications for prehospital administration of whole blood?	Mean 3.53 CI 3.27–3.80	Mean 4.19 CI 3.84–4.54	0.001
How comfortable are you knowing the contraindications for prehospital administration of whole blood?	Mean 3.19 CI 2.89–3.49	Mean 4.16 CI 3.77–4.55	<0.001
How comfortable are you with the procedure to administer whole blood in the field?	Mean 3.11 CI 2.82–3.41	Mean 4.13 CI 3.75–4.51	<0.001
How comfortable are you with prehospital blood administration in pediatrics?	Mean 3.04 CI 2.73–3.34	Mean 3.97 CI 3.58–4.35	<0.001
How comfortable are you with obtaining verbal consent from a patient for whole blood administration? Assume the patient has capacity.	Mean 3.85 CI 3.60–4.10	Mean 4.35 CI 3.98–4.73	0.007
How comfortable would you be handling a situation in which a trauma patient meets the criteria for blood but refuses due to their religious beliefs?	Mean 3.77 CI 3.49–4.10	Mean 4.19 CI 3.82–4.57	0.05
How easy do you think it will be to integrate whole blood administration into your typical care of a patient in hemorrhagic shock?	Mean 3.60 CI 3.39–3.82	Mean 3.94 CI 3.62–4.25	0.07
Was this course too long, just right, or too short?	---	Too long = 3 (9.7%) Just right = 28 (90.3%) Too short = 0 (0.0%)	---
Would you recommend this course to someone else?	---	Yes 96.8% [30/31] No 3.2% [1/31]	---

*CI*, confidence interval.
